# Author Response to Peer Reviews of “A Machine Learning Explanation of the Pathogen-Immune Relationship of SARS-CoV-2 (COVID-19), and a Model to Predict Immunity and Therapeutic Opportunity: A Comparative Effectiveness Research Study”

**DOI:** 10.2196/24739

**Published:** 2020-10-19

**Authors:** Eric Luellen

**Affiliations:** 1 Bioinformatix Interlochen, MI United States

**Keywords:** infectious disease, SARS-CoV-2, COVID-19, public health, immunity: vaccinations, therapeutics, stem-cell growth factor-beta

*Author response to peer review reports for “A Machine Learning Explanation of the Pathogen-Immune Relationship of SARS-CoV-2 (COVID-19), and a Model to Predict Immunity and Therapeutic Opportunity: A Comparative Effectiveness Research Study.”*

## Response to Round 1 Reviews

Regarding reviewer G’s feedback that the abstract read like bullets to a white paper and was stylistically different, and inferior, to the flow of the main paper, the abstract was revised to be more of a narrative while honoring the mandated structured subheadings. Moreover, the reviewer’s suggestion to include limitations and potential socioeconomic impacts of the results similarly improved the impact of the paper and contextualized its findings. The author is grateful for this insightful feedback because it helped improve the readability of the abstract, and may encourage more researchers, practitioners, and journalists to read the paper. 

Regarding reviewer H’s feedback to be consistent with scientific nomenclature (SARS-CoV-2 or the more colloquial COVID-19), the paper was revised to note the alternative colloquial term once in the title and once in the text, and corrected all entries to the more medically and scientifically correct name, SARS-CoV-2. Again, the author is grateful for this constructive criticism because, in addition to consistency, it may impact readers’ inferences about the training of the author and scientific accuracy of the paper and results.

